# Did States With More Social Capital Pre-pandemic Offer Mental Health Protection During the COVID-19 Pandemic? A Cross-Sectional View

**DOI:** 10.3389/fpubh.2022.947569

**Published:** 2022-07-18

**Authors:** Kim Nichols Dauner, Neil A. Wilmot

**Affiliations:** Department of Economics and Health Care Management, Labovitz School of Business and Economics, University of Minnesota Duluth, Duluth, MN, United States

**Keywords:** mental health, social capital, COVID-19, place-affect, states (of the U.S.)

## Abstract

**Background:**

Social capital is a well-known health determinant with both relational and geographic aspects. It can help mitigate adverse events and has been shown to impact behaviors and responses during the COVID-19 pandemic. Mental health has declined during the COVID-19 pandemic, and social capital, may serve to buffer those declines.

**Methods:**

Building from this, we assessed whether pre-pandemic social capital and contemporaneous social policy, which included indicators of social trust, civic participation, and presence of mask mandates, affected pandemic mental health, measured as the percent of the population experiencing symptoms of depression and anxiety at the state level.

**Results:**

Generalized social trust and state mask mandates were significantly associated with lower levels of depression and anxiety. Conversely, states with greater civic engagement prior to the pandemic experienced more anxiety and depression.

**Conclusions:**

Findings suggest that existing social capital, particularly social trust, may protect against anxiety and depression and contribute to community resilience during times of adversity. States should invest in policies and programs that increase social trust.

## Background

Social capital is a multilevel health determinant incorporating aspects of individual social networks, participation in civic life, and contextual factors related to the social and economic organization of neighborhoods, organizations, or communities, together which facilitate coordinated actions to improve society (1). Social capital, in a myriad of different conceptualizations, is positively associated with lower individual mortality (2, 3), improved self-rated health (4, 5), and better general mental health at both the individual (6) and state levels (7). With regard to depression specifically, although some have found no association between individual level social capital and major depression once prior depression is accounted for (8), others have found a negative association between various measures of social capital and/or social trust and depression among various populations including minority women with children (9), adult workers (10), youth at the cusp of adulthood (11), and older adults (12, 13).

The social capital in one's environment, whether that be within a person's community or workplace, impacts health, independent of individual measures of social capital; furthermore, this relationship holds across a variety of settings, study designs or health outcomes (14). Put another way, social capital is both geographical and relational (15, 16). Thus, when it comes to the measurement of social capital, measures are correspondingly situational. Across the literature, surveys of people focus on the constructs of trust, social participation, and reciprocity and these seem to have the strongest relationship with health outcomes. To reflect geography, researchers aggregate measures of people, measure densities of civic organizations, or explore policy dimensions and how they affect health (17). In a county-level spatial analysis, Yang (18) found regional clustering of mental distress and a negative relationship between mental distress and social capital. In addition, they found strong spillover effects from the social capital in adjacent counties; and suggest regional collaboration to improve social capital. These findings contribute to our understanding of the spatial nature of social capital and how it can impact mental health.

Szreter and Woolcock (19) make the theoretical case for the importance of societal relationships and power dynamics in considering the impact of social capital on health outcomes by emphasizing the relational nature of public health, normative behaviors, and how citizen's norms and participation in civic society shape and make policy. Their theory centers social capital in the practices and policy of a place, as well as how the people of a place relate to and interact with those with policy power. We find this particular framework relevant here as we are investigating social capital and health at the unit of the state.

As well, there is increasing recognition that social capital is a key factor in mitigating the impacts of adverse events (20). Mental health has declined over the course of the COVID-19 pandemic (21) with recent research elevating its impact to a global traumatic stressor (22). Meanwhile, there is a growing body of literature attempting to unravel the impact of social capital on the COVID-19 pandemic. During the early days of the pandemic, both individual and policy precautions against infection, such as decreasing trips outside the home (23), wearing a mask (24) and implementing a mask policy (25) were better in areas with high social capital. Increased social trust was associated with greater compliance with self-quarantine regulations in Israel (26). Social capital also appeared to play a role in reducing risk of infection (27) and prompting government response (28). Conversely, social vulnerability has been associated with increased COVID-19 cases and deaths (29). However, it is not known how a location's pre-pandemic social capital impacted mental health during the pandemic. To provide the necessary evidence we capitalize on data from the Centers for Disease Control's Pulse Survey on population mental health during the pandemic, and interstate variability in mental health outcomes and social capital indicators and assess whether state-level pre-pandemic social capital and contemporaneous mask policy, as a type of social trust, affected pandemic mental health. We hypothesize that the dimensions of social capital will confer a protective effect on population level mental health.

## Methods

The study was cross-sectional, at the state level. We used publicly available data from all 50 U.S. states, along with the District of Columbia, compiled from several sources. The dependent variable (*MH*) was the percent of persons in a state reporting symptoms of anxiety or depression on the Centers for Disease Control's Pulse Survey during the period ending February 15, 2021 (30). Chosen due to its contemporary nature, there does not appear to be anything statistically special or unique about this period. The average state reporting level is within 0.5 standard deviation units from the average value of the 27 periods beginning April 23, 2020.

Independent variables on state-level social trust and civic participation precede the pandemic and were obtained from the United States Congress Joint Economic Committee's The Geography of Social Capital in America project (31). Social trust (*social*) was measured as the percentage of the population that “trust all or most of their neighbors”. This is a primary measure frequently used to compare social trust internationally (32). Civic participation (*civic*) was an index aggregating eight individual measures (number of membership organizations per 1,000; religious and non-religious non-profits per 1,000; and percent of population who in the last year had: attended a meeting to discuss politics, participated in a demonstration, volunteered for a group, attended a public meeting, worked with neighbors to improve something, and served on a committee or as a group officer). This index is standardized so its mean is zero and the standard deviation equals one. A third indicator of social capital was whether the state had a *mask mandate* (coded as no mandate, situational, or sweeping) (*mask*) in effect as of February 26, 2021 (33). We chose this indicator recognizing the relationship between social trust and government performance when large-scale collective action is needed, as was the case during the pandemic (34).

We also controlled for state-level population characteristics that are known to impact mental health and policy-making power (19), and obtained these data from the Census Bureau (35). Characteristics included percentage of the population below the poverty level (*poverty*), percentage of the state's population over age 65 (*pop65*), and percentage of the state's population that is Black or African American (*black*). State unemployment rates were obtained from the Federal Reserve Economic Database and used to calculate the difference between the unemployment rate *(*Δ*unemp*) in February 2021 compared to January 2020 (36). We controlled for state level pre-pandemic mental health (*preMH*) by using age-adjusted percentage of adults reporting 14 or more days of poor mental health per month in the state in 2018 (37). Finally, we included the number of covid cases (per 100,000) (*covidper*), as reported by the Center for Disease Control Data Tracker, concurrent with the measure of anxiety (38). Based on the work of Cai et al. (39), individuals living in states with higher COIVD incidence would be expected to experience anxiety and depression at higher rates.

Linear regression was used to analyze the effect of the social capital variables on reported anxiety and depression. Estimation, based on the fifty states and the territory of D.C., used the following regression equation:


(1) $$\begin{array}{r} \begin{array}{c} MH_{i}=\beta_{0}+\beta_{1}social_{i}+\beta_{2}civic_{i}+\beta_{3}mask_{i}\;+\\ \;\beta_{4}\Delta unemp_{i}+\beta_{5}poverty_{i}+\beta_{6}pop65_{i}\;+\\ \;\beta_{7}black_{i}+\beta_{8}preMH_{i}+\;\beta_{9}covidper_{i}\;+\varepsilon_{i}. \end{array} \end{array}$$

## Results

**Figure 1:**
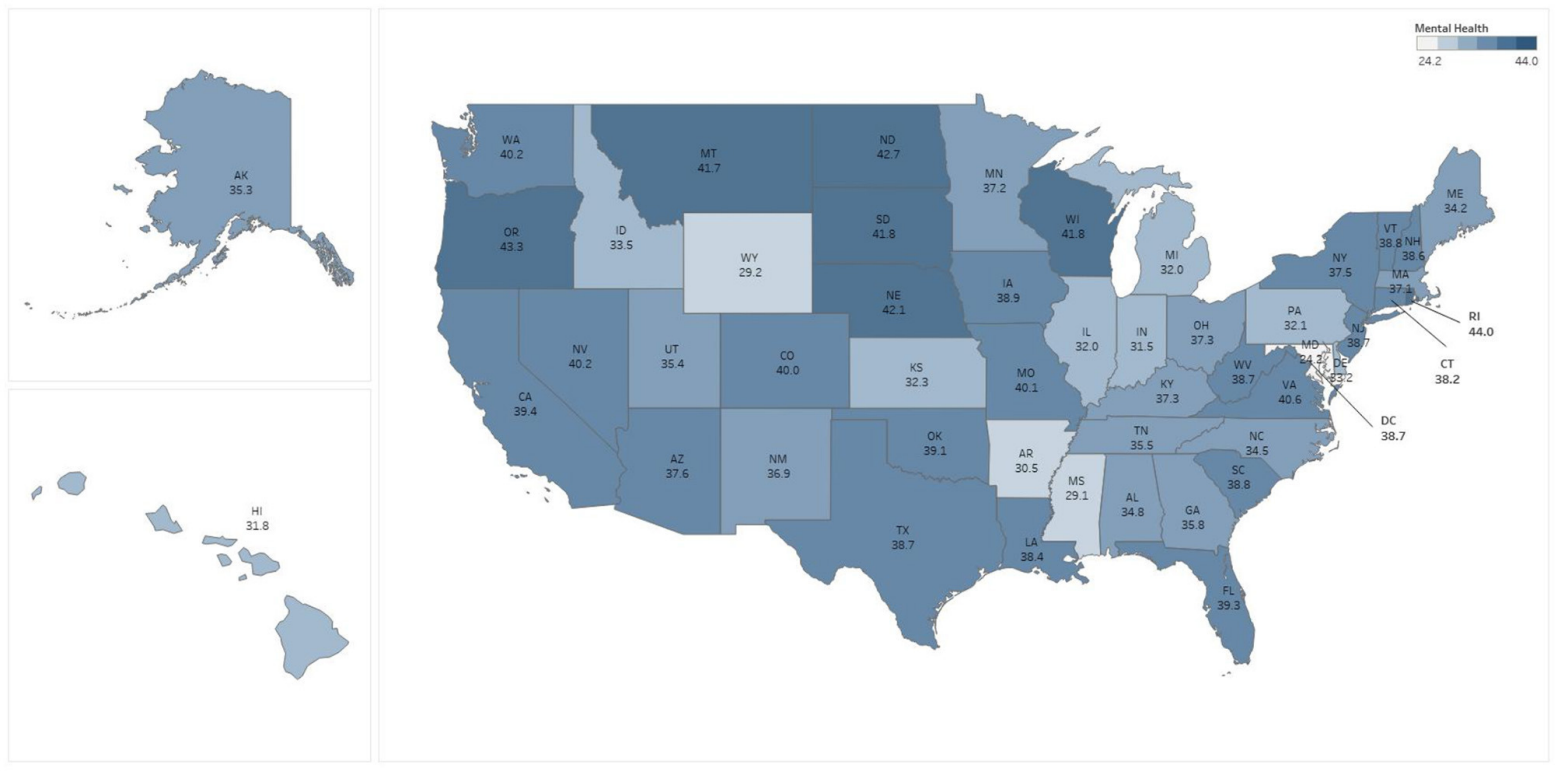
Percent of persons reporting symptoms of anxiety or depression by state, February 2021.

**Figure 2:**
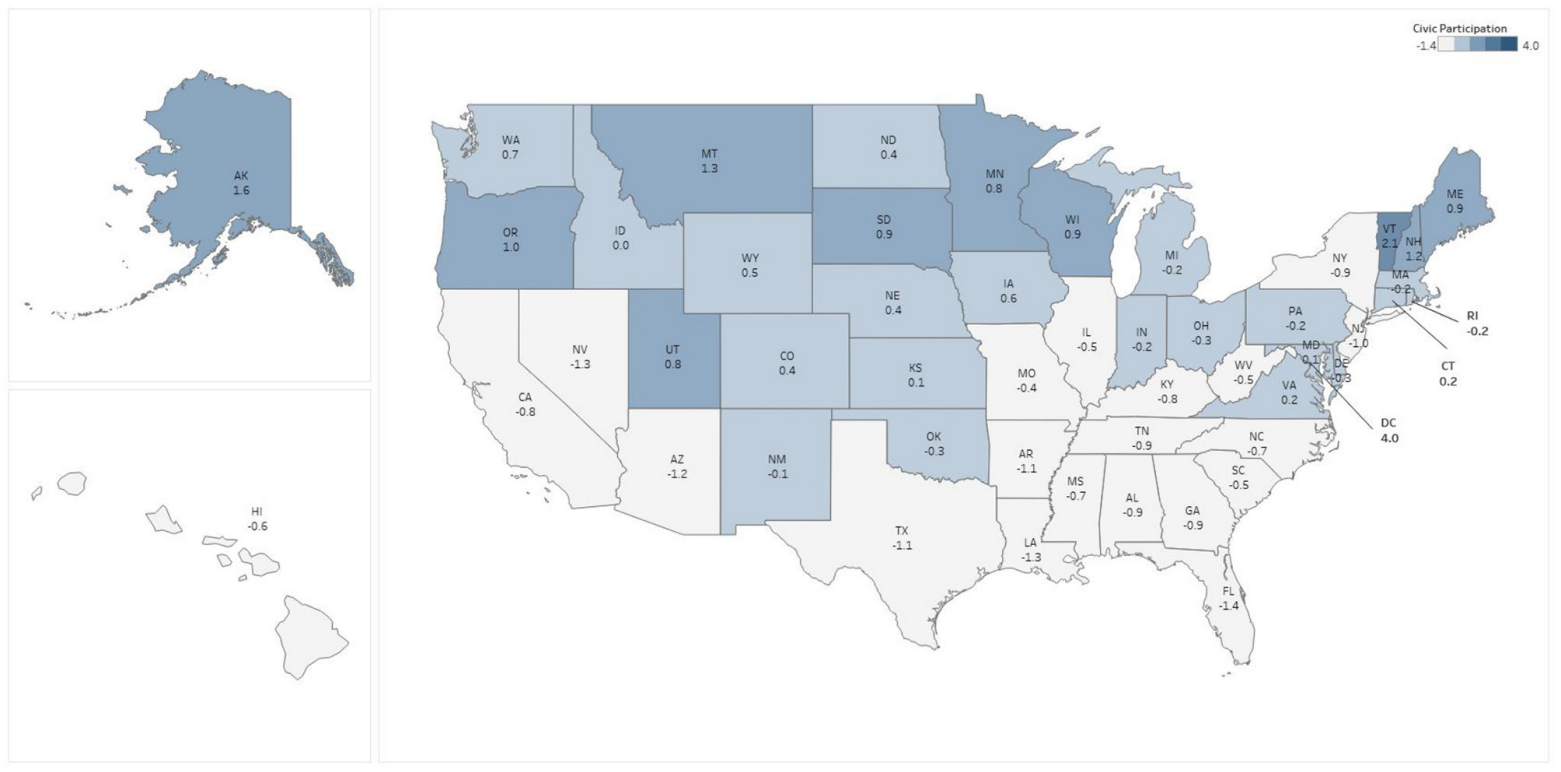
Pre-pandemic civic participation by state.

**Figure 3:**
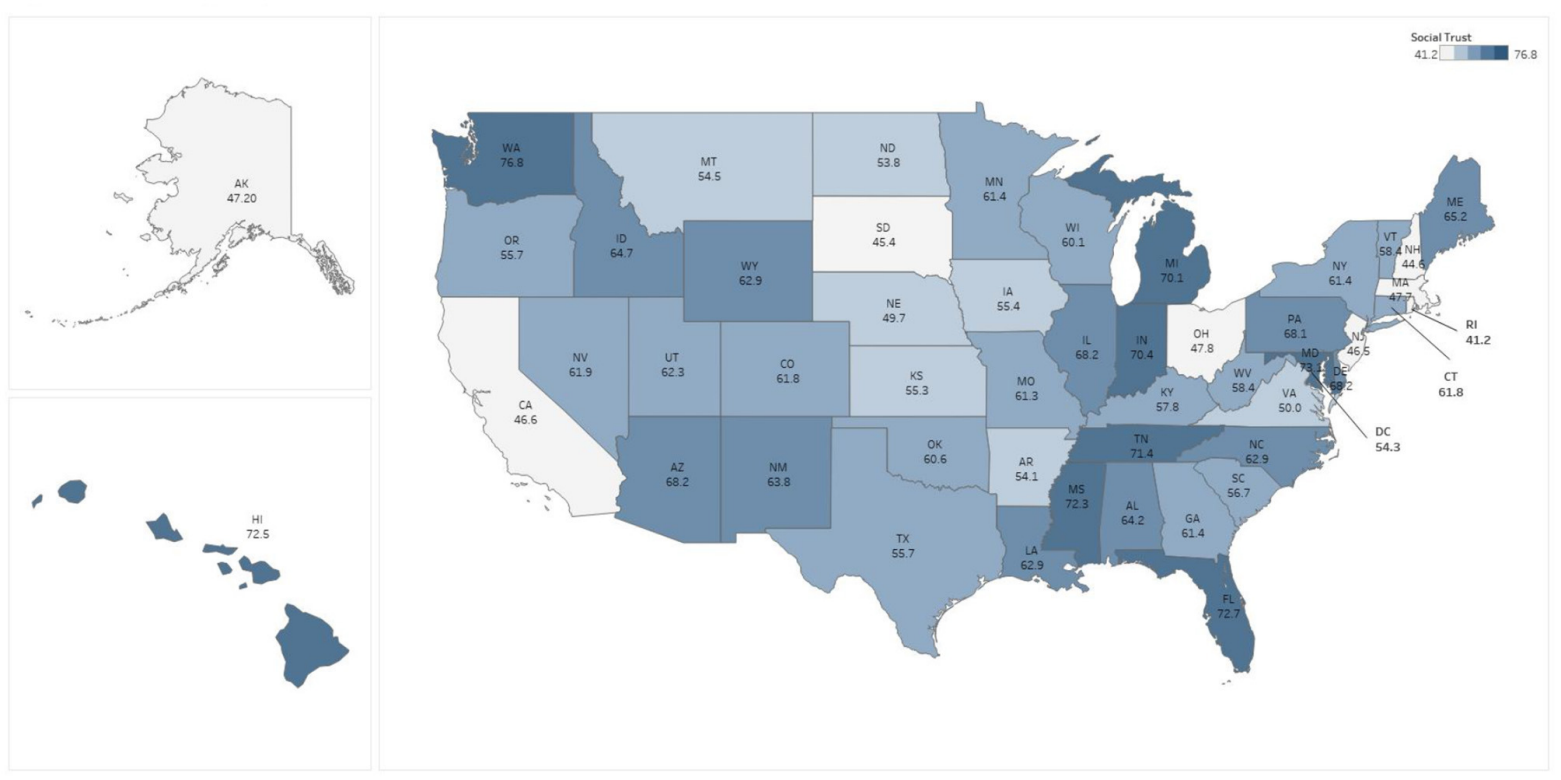
Pre-pandemic social trust by state.

State level percentages of persons reporting symptoms of anxiety and depression ranged from a low of 29.2% (Wyoming), to a high of 45.1% (Oregon), with a median value of 38.7% (Figure 1). Scores for the civic participation index ranged from a high of 3.97 in the District of Columbia to a low of −1.38 in Florida; 20 states had civic participation levels below the mean, with the remaining 30 states and the District of Columbia performing above the mean (Figure 2). Levels of perceived social trust varied considerably across the U.S. and ranged from a high of 76.8% reporting that they ‘trust all or most of their neighbors’ in Utah, to a low of 41.2% in Nevada (Figure 3). As of February 26, 2021 11 states had no mask mandate whatsoever, six had a situational mask mandate, and the remaining 33 states and the District of Columbia had a universal and mandatory mask mandate. We note that the pairwise correlation among social trust and mask mandate variables is low (0.12), as is the correlation among civic participation and the mask mandate (0.07). A correlation matrix for all the predictor variables is available as Supplementary Table S1.

Regression results, utilizing robust standard errors, are presented in Table 1. At the state level, the estimate for social trust is negative and significant, indicating that states where citizens report higher levels of trust among neighbors, had fewer individuals reporting symptoms of anxiety or depression in February 2021. Likewise, the effect of a state face mask policy is negative and significant, meaning that such policies are predictive of lower levels of anxiety and depression. In contrast, the coefficient on the civic participation index is positive and significant at 10%, which suggests that communities with greater civic engagement prior to the pandemic experienced more anxiety and depression. While also significant at the 10% level, we note the positive coefficient on the percent of the population over the age of 65, with larger percentages predicting higher levels of anxiety and depression. Additionally, one might consider the concurrent number of COVID cases (per 100,00 residents) as an important determinant of the level of anxiety or depression in a state. As shown in Table 1, the effect is not significantly different from zero.

**Table 1:** Regression results for state level symptoms of anxiety and depression in U.S. adults, U.S. household pulse survey.

**Variable**	**Mean**	**SD**	**Coefficient**	***p-*** **Value**	***VIF***
Mental health	38.4	3.42	–	–	
Social trust	59.8	8.64	−0.2432^***^	0.005	1.12
Civic participation	0.00	1.0	0.8974^*^	0.064	1.56
Mask mandate	1.4	0.83	−1.2705^**^	0.050	1.69
Δ unemployment	2.0	1.51	0.6121	0.153	2.00
Poverty	12.2	2.68	0.1238	0.619	2.29
% Population over 65	16.9	2.02	0.3990^*^	0.091	1.34
% Population Black	26.0	14.87	0.0382	0.370	1.21
Pre-pandemic mental health	13.3	2.21	−0.1150	0.666	2.76
COVID per capita	9,332.4	2,438.56	−0.003	0.198	1.11
Constant	–	–	47.1619^***^	0.000	
R-square	–	–	0.4113	–	

Based upon robust standard errors, ^***^, ^**^ and ^*^ indicate significance at the 1, 5 and 10% respectively.

The findings offer support for our hypothesis that higher levels of social capital will translate to mental health protection (lower levels of anxiety and depression). While social trust and masking policies seemed to confer some protection for mental health, the findings for civic participation are more nuanced. To ascertain the robustness of these results, numerous versions of models nested in Equation (1). were investigated. The results of this exercise point to the consistency of the coefficient estimates and significance for both social trust and the mask mandate. Within these models, while the civic participation coefficient experienced greater variability, it remained positive and generally significant at the 10% level.

## Discussion

The findings provide a first look into states' social capital and how that social capital may confer mental health protection during the COVID-19 pandemic. We found that states with higher levels of social trust pre-pandemic tended to also have fewer persons experiencing symptoms of anxiety and depression, suggesting that their neighborly relations and sense of community may mitigate these symptoms. As expected, state mask mandates were associated with lower percentages of persons reporting anxiety and depression and could indicate that citizens feel less anxiety knowing the state is actively trying to prevent virus spread. Our findings fit with research by Brodeur and colleagues who found greater decreases in mobility in high-trust counties after stay-at-home orders were implemented (40).

States with higher levels of pre-pandemic civic participation reported greater symptoms of anxiety and depression during the pandemic, although this finding should be taken with a note of caution since it was only significant at the 10% level. While somewhat counterintuitive, it could be a reflection of how the pandemic has limited social interaction, leading to elevated occurrences of anxiety and depression among the residents in those states and fits with other research in this area (28). Our findings compliment prior research on the impact of social capital during the pandemic. We also found that higher percentages of persons over the age of 65 was related to increased anxiety and depression, which may reflect the increased risk of hospitalization and death due to COVID-19 in this age group (41). Finally, these aspects of social capital are significant, even while controlling for pre-pandemic mental health.

This is important as both COVID-19 and mental health concerns persist. Overall, there was wide variation in social trust across states with a 35% difference between states with a little and a lot of social trust. Civic participation also varies, but appears to be lower in the South and Southwest. Such differences between states, indicate that both social trust and civic participation are changeable, though more research is needed into the specific policies and social supports that results in higher social trust or greater civic participation.

Our findings support the importance of existing area level social capital as a health protective factor mitigating adversities associated with the COVID-19 pandemic (27–29). In particular, social trust may protect against anxiety and depression and contribute to community resilience during times of adversity. Our findings are supported by others work in this area. Donnelly and Farina (42), also using Pulse data, found the provision of economic support - Medicaid, unemployment insurance, and suspended utility shut offs - provided a buttress against the impact of household income shocks on mental health. Similarly, our research supports the notion that social policies, many of which are enacted and enforced at state level, can support mental health. In addition to mask policies suggestions to increase social capital include increasing institutional effectiveness, accountability and transparency, and responding to citizen concerns (43). There are a number of specific actions states could take to increase social capital. These include reconnecting Americans to work, improving investment in youth, and making it more affordable to raise a family (31). States could help incentivize and/or finance partnering among human service agencies, faith-based organizations, mentoring programs, and peer and family support programs (44). Likewise, states could invest in community-hospital partnerships that hold promise for increasing social capital and responding to depression (45). States can also leverage federal financing opportunities that show potential to better civic infrastructure and increase social capital (46).

This research has some limitations. One limitation of the research is that the data are cross sectional and cannot establish causality, albeit two of the independent variables are captured from an earlier time point compared to the dependent variable. We only measured three aspects of social capital and assumed that the social capital variables were relatively static during the pandemic; however, some research has found declining trust over the course of the pandemic (21). Nevertheless, we use distinct measures—aggregate data, behavioral, and policy—that get at different constructs of social capital. Future research should continue to explore how mental health has fared over the course of the pandemic, and the role of social capital on mental health and resiliency. Similarly, this research cannot rule out the presence of important but unmeasured variables. Because this study is ecological in nature and variables are at the state level, we cannot make conclusions about the relationship between individual social capital and mental health during the pandemic. Similarly, spillover effects may occur between states; however, given distinct policy and social environments in individual states, we don't feel there would be strong state-level spillover effects. One final caveat is that social capital may change as a result of the pandemic. Indeed, nascent research suggests that over the course of the pandemic social capital has changed in different ways in different populations (47, 48). More research is needed to ascertain the impacts of the pandemic on social capital, in which populations or geographies those changes occurred, and the permanence of those changes.

## Data Availability Statement

The datasets presented in this study are publicly available and can be found in online repositories. The names of the repository/repositories can be found in the article/Supplementary Material.

## Author Contributions

KD designed the study. NW analyzed data. KD wrote the initial draft of the manuscript. Both authors identified data sources, interpreted the analysis, edited subsequent drafts of the paper and have read, and approve the final version.

## Conflict of Interest

The authors declare that the research was conducted in the absence of any commercial or financial relationships that could be construed as a potential conflict of interest.

## Publisher's Note

All claims expressed in this article are solely those of the authors and do not necessarily represent those of their affiliated organizations, or those of the publisher, the editors and the reviewers. Any product that may be evaluated in this article, or claim that may be made by its manufacturer, is not guaranteed or endorsed by the publisher.
