# Supplementary material for: Did States With More Social Capital Pre-pandemic Offer Mental Health Protection During the COVID-19 Pandemic? A Cross-Sectional View
Source: Front Public Health. 2022 Jul 18;10:947569. doi: 10.3389/fpubh.2022.947569 (PMC9339967; doi:10.3389/fpubh.2022.947569)
Supplement: Supplementary file 1 [file Table_1.docx]

**Did States with More Social Capital Pre-pandemic Offer Mental Health Protection During the COVID-19 Pandemic? A Cross-sectional View**

Supplementary Materials

The table below presents the pairwise correlation between the predictor variables included in the regression analysis presented in the manuscript.

Table S.1. Correlation Matrix

|  | Social Capital | Com. Health | Mask  Man. | ∆ Unemp. | Poverty | % Pop over 65 | % Pop Black | Pre-pan Mental Health |
| --- | --- | --- | --- | --- | --- | --- | --- | --- |
| Community  Health | -0.2562 | . | . | . | . | . | . | . |
| Mask  Mandate | 0.0080 | 0.0724 | . | . | . | . | . | . |
| ∆ Unemployment | 0.0192 | -0.2376 | 0.4470 | . | . | . | . | . |
| Poverty | 0.2080 | -0.4088 | 0.1312 | -0.1452 | . | . | . | . |
| % Population over 65 | 0.1615 | -0.1444 | -0.0697 | -0.1012 | 0.0741 | . | . | . |
| % Population Black | -0.0790 | -0.1245 | 0.0647 | 0.0583 | 0.1800 | -0.2590 | . | . |
| Pre-pandemic Mental Health | 0.1466 | -0.2373 | 0.0690 | -0.3361 | 0.6402 | 0.3371 | 0.0299 | . |
| Covid case per  capita | -0.0060 | 0.1100 | -0.0697 | 0.1016 | -0.0566 | 0.0088 | -0.2255 | -0.1093 |
